# Novel Intravenous Nanoemulsions Based on Cannabidiol-Enriched Hemp Oil—Development and Validation of an HPLC-DAD Method for Cannabidiol Determination

**DOI:** 10.3390/molecules30020278

**Published:** 2025-01-12

**Authors:** Agnieszka Sobczak, Piotr Zieliński, Anna Jelińska, Aleksandra Gostyńska-Stawna

**Affiliations:** Chair and Department of Pharmaceutical Chemistry, Poznan University of Medical Sciences, Rokietnicka 3, 60-806 Poznan, Polandajelinsk@ump.edu.pl (A.J.); agostynska@ump.edu.pl (A.G.-S.)

**Keywords:** cannabidiol, hemp oil, intravenous nanoemulsion, DAD-HPLC, validation

## Abstract

Background: Intravenous nanoemulsions (NEs) are gaining attention as potential delivery systems for poorly water-soluble substances like cannabidiol (CBD). This study aimed to develop novel NEs based on CBD-enriched hemp oils and evaluate their physiochemical properties. Methods: The stability of hemp oils enriched with various concentrations of CBD (0.5%, 1.0%, and 1.5%) with and without the addition of α-tocopherol was determined, and the most stable oils were subsequently incorporated into NEs. In order to determine the CBD content in the obtained CBD-enriched oils and NEs, as well as to conduct stability tests, a new HPLC method was developed and validated. Results: The HPLC method demonstrated very good linearity, precision, accuracy, specificity, and robustness, enabling reliable assessment of the quality of newly developed formulations. The formulated NEs were characterized by droplet size of below 200 nm and polydispersity index PDI ≤ 0.14 satisfactory for intravenous application. Conclusion: This research presents a preliminary study on the development of CBD-enriched hemp oil-based NEs that showed promising potential for further investigation. A new HPLC-DAD method was appropriate to register changes in CBD concentration in various matrices, including CBD-hemp oil and intravenous NEs during their preparation and storage. Additionally, the effect of certain emulsifiers used in NE formulations on the course of the chromatographic analysis of CBD was examined, providing valuable insights concerning the application of the provided methodology in future formulation analysis.

## 1. Introduction

In recent years, there has been significant interest among scientists and consumers in one of the main biologically active compounds found in *Cannabis sativa*–cannabidiol (CBD). This compound, next to tetrahydrocannabinol (THC), is the most common and best-studied phytocannabinoid. Despite crossing the blood–brain barrier, CBD lacks psychoactive effects, such as the feeling of intoxication or euphoria typically associated with THC. As a result, it has no potential for abuse and is legal in many countries, which, alongside the diverse range of products available and potential health benefits, contributes to its popularity. Only small amounts of CBD may be present in products based on pure hemp oil obtained through cold-pressing of hemp seeds. These result from contamination of seeds by other plant fragments containing glandular trichomes rich in CBD [1,2]. Significant amounts of CBD are found in products enriched with CBD, e.g., obtained after dissolving pure CBD or hemp flower extract in oil. There are many CBD-infused products available on the market, including cosmetics (emulsions, creams, shampoos), food and beverage enhancers (candies, hemp oil), dietary supplements (oils, soft gel capsules, pastes, plant extract oil drops, oral drops), and medications (Sativex Oromucosal Spray containing CBD + THC, Epidiolex oral solution containing pure CBD, and hemp droughts for smoking or vaporization) [3]. In CBD products, alcohols (e.g., ethanol or polyethylene glycol) or lipids (e.g., hemp oil, sesame oil, coconut oil, olive oil) are used as solvents, which is due to high lipophilicity (logP of 6.3) and low solubility of CBD in water (12.6 mg/mL) [3,4,5]. In addition to low water solubility, CBD is characterized by variable pharmacokinetic profiles, poor stability, and a high extraction with an extensive first-pass effect. The pronounced pre-systemic metabolism results from phase I oxidation, mainly caused by CYP3A4 and CYP2C19, and phase II glucuronidation by UGT1A9 [3]. The above features are the reason for its poor bioavailability. After oral administration, the bioavailability of CBD is approximately 6%, increasing to 25% when co-administered with food lipids. Transmucosal and sublingual administration of CBD may result in a rapid onset of action with reduced first-pass metabolism. However, CBD in the form of sublingual drops showed a similar bioavailability to oral administration, probably due to the swallowing of the drug before its absorption [4,5,6]. Bioavailability after inhalation ranges from 2 to 56% due to different smoking dynamics [5]. After topic application, the compound’s high lipophilicity and suitable molecular weight (314.46 Da) result in CBD remaining in the stratum corneum without penetrating the deeper tissue layers [4].

Because the bioavailability of CBD is low, research is still ongoing on various types of CBD administration and optimization of drug compositions (development of new formulations) [3,4,5]. For years, many studies have been conducted to improve bioavailability and ensure appropriate effectiveness [3]. There is great hope for nanoformulations to become CBD carriers. These include nanosuspensions, polymeric micelles and polymeric nanoparticles, inorganic nanoparticles jelled in cross-linked chitosan-based patches, and numerous nanosized lipid formulations. Among the latter, nanostructured lipid carriers, vesicles, self-nanoemulsifying drug delivery systems, nanoemulsions (NEs), and microemulsions are being investigated. Some of the formulation studies with CBD are very preliminary; others, based on known and safe carriers and conventional routes of administration, are at the clinical or preclinical study stage [3,4,5,7,8,9,10]. Intensive research into increasing the bioavailability of CBD is associated with its potential multidirectional biological activity and the possibility of utilizing it in the treatment of various diseases. Preclinical studies demonstrate its anti-inflammatory, antioxidant, analgesic, neuroprotective, and cardioprotective effects. Furthermore, its preventive or disease-slowing activity is being investigated in various conditions, such as cancer, viral diseases, and neurodegenerative disorders [1,11]. At the same time, researchers point out that most people tolerate CBD, and it has a low side effect profile, including mild gastrointestinal problems and drowsiness. However, there are reports that due to the use of high doses of CBD, especially by people with liver damage or taking hepatotoxic drugs at the same time, there is a risk of hepatotoxicity and drug interactions [12,13].

On the other hand, contrary to the above-mentioned potential hepatotoxicity of CBD, the hepatoprotective effect has also been reported. The endocannabinoid system is recognized as a complex signaling biochemical pathway closely related to metabolic disorders. It is a crucial target for treating non-alcoholic fatty liver disease (NAFLD). Despite a lack of conclusive clinical trials, observational and pre-clinical studies highlight the putative benefits of phytocannabinoids on liver steatosis through multiple pathways [14]. For this reason, intensive research is ongoing on the use of CBD in the treatment of liver diseases, such as alcoholic liver disease, liver fibrosis, hepatocellular carcinoma, chemical liver damage, viral infection, autoimmune hepatitis, and liver ischemia–reperfusion injury, and above all, NAFLD [11]. NAFLD, which affects nearly 30% of the general adult population, most often occurs in patients with metabolic syndrome: obesity, insulin resistance, diabetes, hyperlipidemia, and hypertension [11]. Other factors causing NAFLD may be a fatty and high-calorie diet, drug-related medications, previous abdominal procedures, etc. Steatosis occurs also in patients suffering from intestinal failure-associated liver disease (IFALD), often together with cholestasis. IFALD is a result of long-term parenteral nutrition and intestinal injury. The process of IFALD development is multifactorial, and it is related, among others, to the lack of antioxidants, the presence of phytosterols in the lipid emulsion, and inappropriate composition of parenteral emulsion admixture (unsuitable content in essential fatty acids, inappropriate ratio of ω-6/ω-3 fatty acids, and too high glucose concentration) [15].

Although CBD is usually administered orally or by inhalation in humans, we decided to develop the intravenous dosage form, which allows the administration of this agent to parenterally fed patients and, at the same time, provides the protection of CBD against degradation in the gastrointestinal tract and the first-pass metabolism of the liver. In this study, we estimated the stability of hemp oils enriched with various concentrations of CBD (0.5%, 1.0%, and 1.5%) with and without α-tocopherol, and the most stable oils were applied to NEs. In order to perform the quantitatively CBD content, we developed and validated a new high-performance liquid chromatography with diode-array detection (HPLC-DAD) method that allows monitoring of the CBD content in pure raw material, CBD oil, and intravenous NEs. Our research included determining the CBD content in the CBD-enriched hemp oils and NEs, establishing their stability in selected conditions, as well as the physical characteristics of the obtained delivery system.

## 2. Results and Discussion

Intravenous NEs have garnered increasing attention as delivery systems for poorly water-soluble substances such as CBD. These systems address several challenges associated with the administration of such compounds. Intravenous injection offers significant advantages over oral delivery, currently the primary route for CBD in clinical settings. Unlike oral administration, intravenous drug delivery provides 100% bioavailability and reproducible pharmacokinetics by bypassing the gastrointestinal absorption barrier [16]. Additionally, NEs can reduce injection-related pain by minimizing the use of synthetic excipients, such as propylene glycol, commonly required as solubilizers in non-emulsion intravenous formulations [17].

**Figure 1:**
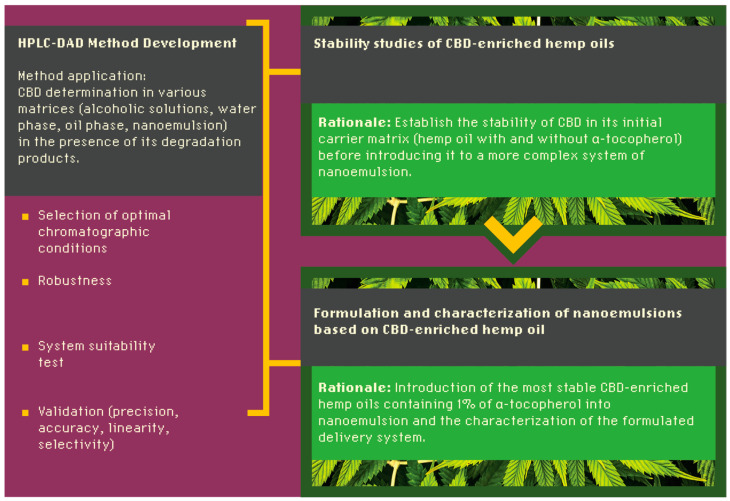
Scheme of study design (CBD–cannabidiol).

In this study, we developed a novel CBD-enriched hemp oil-based NE. Hemp oil served as a carrier for incorporating CBD into the formulation. Given the inherent instability of CBD, we decided to investigate the potential of α-tocopherol, an antioxidant, to enhance its stability. The initial phase of the study involved evaluating the stability of refined hemp oils enriched with varying CBD concentrations (0.5%, 1.0%, and 1.5%) with and without the addition of α-tocopherol (Section 2.1). The subsequent stage focused on preparing NEs using the most stable oil identified from the stability tests. These NEs were then characterized in terms of key parameters, including mean droplet diameter (MDD), polydispersity index (PDI), peak size, zeta potential (ZP), stability, and CBD content (Section 2.2). A central research component was developing a robust quantitative method for determining CBD content, across various matrices such as alcoholic solutions, water phase, oil phase, and NEs (Section 2.3). This method enabled a comprehensive evaluation of both the stability of CBD and the quality of the developed NEs. The study design, including the individual research stages, is visually summarized in Figure 1.

### 2.1. Stability Studies of CBD-Enriched Oils

Cannabidiol is highly susceptible to environmental factors such as temperature, light, and oxygen, leading to its decomposition during analytical procedures, manufacturing, storage, and even after administration but before intestinal absorption [18,19].

Literature reports confirm the solvent-dependent nature of CBD degradation, with faster degradation observed in aqueous media than in ethanol (t_95_ = 7 days at 25 °C vs. t_95_ = 117 ± 18 days). Under simulated physiological conditions (pH 7.4 and 37 ± 2 °C), up to 10% degradation of CBD was observed within just 24 h. The choice of organic solvent (methanol, acetonitrile, and n-hexane) also significantly influences the degradation rate, particularly under light exposure [20].

Based on studies of CBD stability in ethanol solutions across temperatures ranging from 25 to 70 °C, Fraguas-Sánchez et al. determined that temperature is another critical parameter. Using the Arrhenius equation, the activation energy for the degradation process under these conditions was determined to be 92.19 kJ/mol, confirming the compound’s susceptibility to temperature-induced decomposition. CBD was found to be unstable at room temperature (t_95_ = 117 ± 18 days), but stable for at least 12 months at 5 °C (calculated t_95_ = 4.8 years) [19]. These findings emphasize the importance of low-temperature storage to maintain CBD integrity.

Moreover, CBD has been observed to degrade when exposed to oxidizing agents and light, with photodegradation occurring more prominently in the presence of other destructive factors such as increased temperature, oxygen, and some solvents. Studies by Kosović et al. demonstrated significant degradation of CBD in solid powder form and when dissolved in sunflower oil under various storage conditions, highlighting the impact of temperature and humidity on stability [18]. Kosović et al. conducted the stability tests of CBD in the form of a solid powder and also dissolved in sunflower oil under 25 ± 2 °C/60 ± 5% RH and 40 ± 2 °C/75 ± 5% RH, in open and closed vials, protected from light. CBD in the form of solid powder was decomposed under the tested conditions at a level of approximately 10% over one year [18]. However, a decrease in the amount of CBD in the form of oil occurs much faster, especially in open vials. In open vials at 40 ± 2 °C/75 ± 5%RH, between the 90th and 180th day, significant degradation was observed, with a complete degradation of CBD after approximately 270 days. In the case of closed vials, the degradation was slightly slower (after 180 days, there was a significant CBD loss of 16.5%, and after one year, about 76%) [18].

In our research, we decided to use hemp oil as a carrier for incorporating CBD into the NE. To improve CBD stability, we investigated the potential protective effects of α-tocopherol as an antioxidant.

Stability assessments were conducted under various conditions: at room temperature (with and without light exposure), refrigeration (4 ± 2 °C), and accelerated aging in a climatic chamber (60 ± 2 °C, 75 ± 5% RH, without light exposure). The results (Table 1) showed that CBD-enriched oils stored at room temperature for 2 months experienced degradation ranging from 4.6% to 16.4%, depending on the concentration and exposure conditions. In contrast, refrigeration significantly reduced CBD loss (degradation ranging from 2.8% to 4.9%), demonstrating the critical role of temperature in extending CBD shelf life. Under accelerated aging conditions (60 ± 2 °C and 75 ± 5% RH, in closed vials), significant degradation was observed within 4 weeks, with higher degradation rates in oils without α-tocopherol compared to oils containing the antioxidant (the differences in loss were 19.4%, 18.3%, and 7.2% for 0.5%, 1.0%, and 1.5% CBD oil, respectively). These results confirm the potential protective effect of α-tocopherol.

These findings align with those reported by Kosovic et al. (degradation under increased temperature and relative humidity of 75%), although the observed differences in degradation levels can be attributed to the different storage conditions (60 °C vs. 40 °C) [18].

The CBD content in the oils before and after thermal sterilization was also determined (121 °C, 20 min). In the case of oils without the addition of α-tocopherol, a decrease in content of up to 11% was observed, in the case of CBD-enriched oils containing α-tocopherol, no significant change (more than 4%) in concentration was observed (Table 1). The results of this study highlight the critical role of storage conditions and the addition of antioxidants in preserving the stability of CBD-enriched oils. α-Tocopherol demonstrated considerable potential in mitigating degradation, making it a promising stabilizing agent for CBD formulations.

### 2.2. Development and Characterization of Nanoemulsions

To enable the intravenous administration of CBD, we incorporated the most stable hemp oils, enriched with CBD at concentrations of 0.5%, 1.0%, and 1.5%, and supplemented with 1% α-tocopherol, into NEs. These formulations underwent comprehensive evaluation, including particle size determination and ZP evaluation, to confirm compliance with the pharmacopeia requirements regarding MDD [21] and assess the predicted stability of the oil-in-water system. Both parameters were measured at the three critical stages: immediately after preparation, following filtration sterilization, and after 3 weeks of refrigerated storage at 4 ± 2 °C, as a preliminary stability assessment. The characterization of prepared NEs is presented in Table 2.

**Figure 2:**
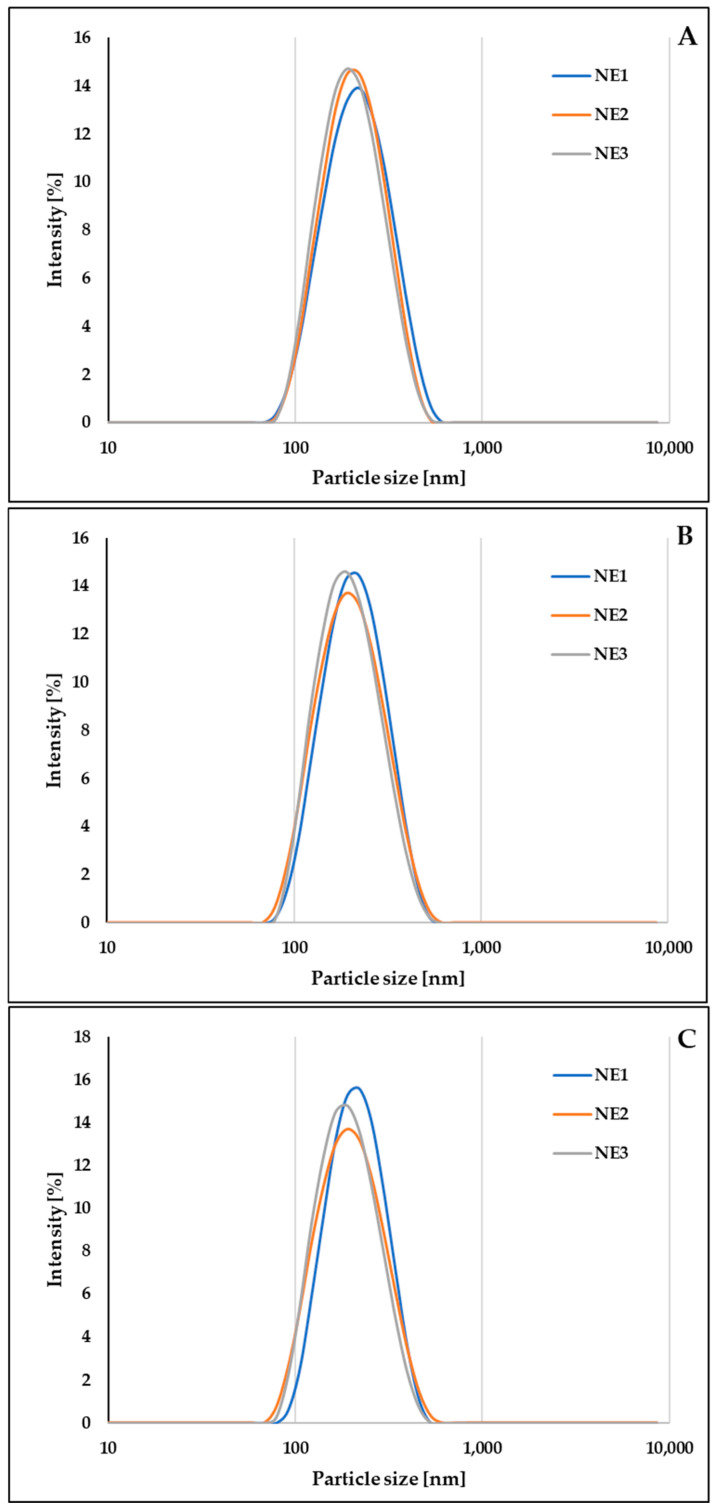
The droplet size distribution by the intensity of CBD-loaded NEs immediately after preparation (**A**), after filtration sterilization (**B**), and after 3 weeks of storage in the refrigerator (**C**).

All prepared NEs were characterized by MDD below 200 nm, narrow particle size distribution (PDI of less than 0.15), and ZP ranged from −27.8 ± 0.4 to −51.2 ± 0.7. The droplet size distribution by the intensity of the obtained NEs on the day of preparation, after filtration sterilization, and 3 weeks of storage is presented in Figure 2. The filtration sterilization led to a slight but insignificant decrease in the MDD values (*p* > 0.05). This process slightly affected the ZP values in all samples except NE1, where an increase in ZP value of about 10 mV was observed.

The results showed that all prepared NEs were characterized by MDD values satisfactory for intravenous administration. The United States Pharmacopeia in Chapter <729> [21] indicates that the MDD in the injectable emulsions determined using dynamic light scattering or classical light scattering methods cannot exceed 500 nm. This limit is dictated by the risk of embolism, which results from the diameter of the capillaries in the case of administration of droplets with a larger diameter. Moreover, the DLS method can be applied only if the PDI is lower than 0.7 indicating the sufficient distribution of droplet size [22]. The PDI values determined in all measurements did not exceed 0.15, which, according to Wang et al., is satisfactory for parenteral applications (PDI values up to 0.25 are acceptable) [23]. Comparing the droplet size before and after filtration sterilization, it was shown that this process decreases the MDD values, indicating that the applied sterilization method seems to be satisfactory for obtaining sterile formulation. Nevertheless, another important factor characterizing intravenous NEs is ZP. The filtration sterilization affected the ZP values leading to its changes into higher numbers in the case of NE1, which theoretically means lowering its stability. Nevertheless, the sterilized NEs were characterized by quite low ZP values, measuring less than −28.4 ± 0.2, which aligns with findings reported by other researchers [24,25]. Moreover, our preliminary stability test showed that the storage for 3 weeks in the refrigerator condition only slightly affected the ZP values indicating good stability over time and further confirming the satisfactory physical characteristics of developed NEs for intravenous administration.

In addition, the CBD content in NEs was determined, and its level was assessed at individual stages of formulation preparation. CBD content was assessed in oils and NEs prepared on their basis, NEs after sterilization, and after 3 weeks of storage. The results are presented in Table 2. Differences in the content of CBD in oils and corresponding NEs after preparation are at a similar level for individual NEs. This proves that the efficiency of the NEs production process is high (98.5–101.0%), and the homogenization process did not have a destructive effect on CBD (CBD loss of 0.1 to 1.9% after preparation of NEs). Similarly, a small loss of CBD content accompanies sterilization (0.5–3.0%) and storage (0.2–2.5%) for 3 weeks at 4 ± 2 °C. To sum up, it can be concluded that such factors as homogenization (ultrasonic and mechanical high-pressure), filtration sterilization, and storage for 3 weeks in the refrigerator do not significantly affect the CBD content.

The proposed delivery system demonstrates significant potential, particularly in light of recent studies on intravenous formulations of CBD. For instance, Muresan et al. developed an Intralipid-based NE by incorporating CBD using a propylene glycol–ethanol mixture (9:1, *v*/*v*) into a commercial intravenous NE [26]. Intralipid is a soybean oil-based lipid emulsion stabilized by egg yolk phospholipids and glycerol. The prepared formulation resulted in a MDD of 286.8 ± 13.5 nm with a PDI of 0.135. Biodistribution studies in rats revealed the efficacy of NEs in achieving rapid and sustained CBD delivery to the central nervous system (CNS) after intrathecal administration, with higher brain retention compared to polymer-coated nanoparticles (characterized by an MDD of 121.8 ± 1.1 nm and a PDI of 0.079 ± 0.014). These findings suggest that the NEs developed in our study, with MDDs ranging between 181.0 ± 2.0 and 191.2 ± 1.7 nm, hold significant promise as delivery systems targeting the brain. Similarly, Mobaleghol et al. prepared a NE containing active components of cannabis extract (THC and CBD) for their potential anticancer effects in glioblastoma treatment [27]. Their formulation combines 300 ng/mL of THC and CBD in a 1:1 ratio dissolved in hemp seed oil (1.00% *w*/*w*) with Span 80 (7.82% *w*/*w*), Tween 80 (27.18% *w*/*w* m/m), ethanol (10%), and water, achieved an MDD of 12.7 ± 1.2 nm. This NE effectively reduced tumor volume in rats (~4-fold on day 7 of treatment compared to the control). However, it is important to note that the excipients and their concentrations play a critical role in the safety and efficacy of NE formulations. Safety concerns associated with excipients such as polysorbate 80 (Tween 80) and propylene glycol, as highlighted by Schwartzberg et al. [28] and Lim et al. [29], must be carefully considered. High concentrations of these emulsifiers are associated with adverse effects, including CNS toxicity, hyperosmolarity, hemolysis, cardiac arrhythmias, seizures, agitation, and lactic acidosis in the case of propylene glycol, as well as hypersensitivity reactions, hepatotoxicity, nephrotoxicity, and infusion-site reactions (e.g., pain, erythema, thrombophlebitis) in the case of Tween 80. These findings underscore the necessity of cautious excipient selection, particularly for formulations for chronic or pediatric patients. Given these considerations, it is worth emphasizing that the NE developed in this study, utilizing soybean phospholipids as an emulsifier with an established safety profile and the hepatoprotective effect documented by Gundermann et al., demonstrates significant potential for further clinical applications [30]. While our study primarily focused on the physical and chemical stability of the formulation, the findings suggest that our formulations have the potential to achieve efficient drug delivery across the blood–brain barrier with fewer adverse effects compared to existing formulations.

Nevertheless, further studies confirming the efficiency, safety, and stability of such formulations are needed before testing their therapeutic potential in vivo on animals and humans in clinical trials.

### 2.3. Chromatographic Analysis

#### 2.3.1. Development of HPLC-DAD Method

We developed an HPLC method to characterize the newly formulated NEs based on CBD-enriched hemp oils and to assess the stability of CBD in oils and NEs in selected conditions. The method development comprised two primary objectives: (1) optimizing conditions for CBD quantification in raw materials, in water and ethanolic phase, and CBD-enriched hemp oils in the presence of degradation products and (2) evaluating the method’s applicability in monitoring CBD concentrations during NE preparation and storage.

Chromatographic optimization focuses on selecting the analytical wavelength, stationary phase, mobile phase composition and its flow rate, type of elution (isocratic and gradient), and column temperature. To determine the conditions for the CBD separation from decomposition products and potential impurities/excipients, CBD solutions in various solvents and CBD samples degraded under selected conditions were applied. UV spectral analysis identified λ = 230 nm as optimal for sensitivity and baseline stability. LiChrosorb RP18 column (125 × 4 mm, 5 μm, Merck, Darmstadt, Germany) was selected based on the proven suitability of columns filled with octadecylsilane-bonded silica gel in cannabinoids analysis [31,32,33].

Under the tested separation conditions, a symmetrical CBD peak with an optimal retention time was obtained using isocratic elution. However, satisfactory separation of the substrate from the degradation products and between them was yet to be achieved. Increasing the proportion of the aqueous part in the mobile phase or lowering the pH resulted in extending or reducing the CBD retention time, respectively; influenced the shape of its peak but did not significantly improve the separation of CBD from its degradation products or the separation of the products from each other. Similarly, adding triethylamine or KCl did not positively affect the separation. Gradient elution using the mixture of acetonitrile and water as the mobile phase was finalized, providing efficient separation of CBD from degradation products. Final conditions included a flow rate of 1.5 mL/min, a column temperature of 40 °C, and an analysis time of 12 min. The established conditions ensured an appropriate shape of the peak, separation, and analysis time.

##### Robustness

Following the requirements of the International Council for Harmonization (ICH Q14), as part of method development, a robustness test was conducted [34]. Robustness testing confirmed method reliability under minor parameter variations, such as column and autosampler temperature, flow rate of mobile phase, injection volume, and analytical wavelength (Table S1, see Supplementary Materials). The observed changes in CBD quantification between standard and changed conditions were ≤1.0%, with peak symmetry coefficients ranging from 0.9 to 1.0, ensuring consistent performance. This optimized HPLC method offers a robust and reliable tool for accurately quantifying CBD and monitoring its stability in complex formulations, ensuring its suitability for quality control in CBD-based products.

#### 2.3.2. Validation of HPLC-DAD Method

The next stage involved verifying the developed method’s suitability for the specified purpose through its validation and performing the system suitability test (SST). Validation was carried out according to the International Council for Harmonization (ICH Q2R2) [35] requirements concerning selectivity, linearity, precision, and accuracy.

##### System Suitability Test

System suitability test is an integral part of analytical procedures to determine the suitability and effectiveness of a chromatographic system before or during the analysis. This test is conducted at the method development stage to establish limits for specific parameters, ensuring the reliability of the results [ICH Q14] [34]. The system suitability test included checking the following parameters: the capacity factor (k’), the number of theoretical plates (N), and the peak symmetry factor, as well as the repeatability of the peak position (t_R_) and peak area. The determined values of selected parameters and acceptance criteria are presented in Table S2. All determined parameters are within accepted ranges. On their basis, it can be concluded that the developed method is suitable and effective for CBD analysis if the selected parameters meet the above acceptance parameters.

##### Selectivity

**Figure 3:**
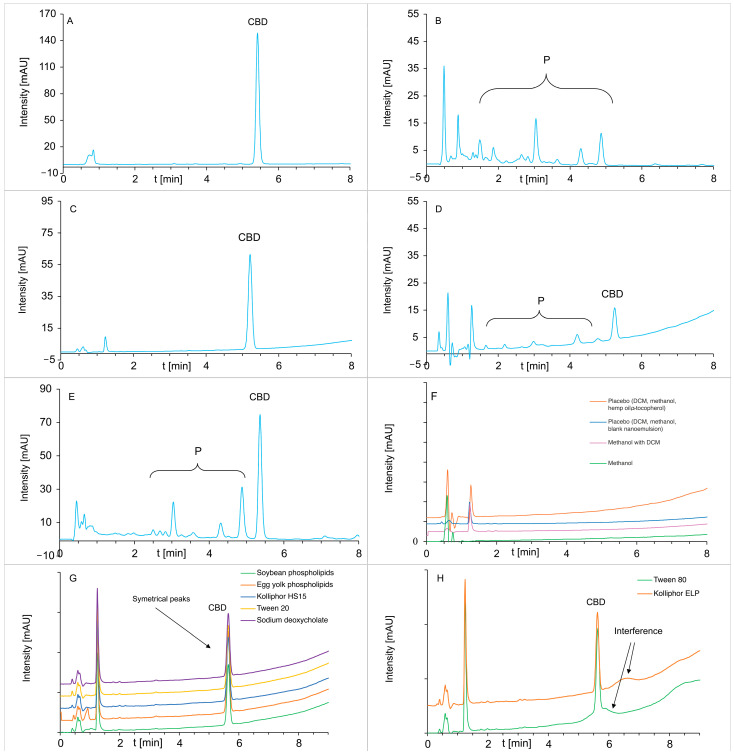
Chromatograms of: (**A**) CBD degraded in a mixture of 0.8% ethanol and an aqueous solution of 1% SLS (T = 37 °C) at t = 0 and (**B**) t = 42 days; (**C**) solution of 1.5% CBD-enriched oil with α-tocopherol in methanol and DCM (30 μg of CBD/mL); (**D**) degraded CBD-enriched oil in 60 ± 2 °C, 75 ± 5%RH, 4 weeks; (**E**) CBD after irradiation in 96% ethanol (T = 25 °C, t = 72 h, intensity 765 W/m^2^); (**F**) Solvents, placebo; (**G**) CBD solutions (4 μg/mL) with various emulsifiers; (**H**) CBD solutions with Tween 80 and Kolliphor ELP. CBD—cannabidiol; P—degradation products of cannabidiol; DCM—dichloromethane.

Using a DAD detector in HPLC analysis, the critical validation step demonstrates the method’s selectivity. The selectivity of the developed method was evaluated by verifying the chromatograms of CBD, blank samples of the various solvents, and the excipients (e.g., hemp oil with α-tocopherol, NEs, emulsifiers, dichloromethane—DCM, ethanol, methanol, sodium lauryl sulfate—SLS, lactose), and degraded CBD in selected matrices. Examples of chromatograms confirming the sufficient separation of CBD from its degradation products, from solvents, and the ingredients of our NEs are presented in Figure 3 and Figure S1.

To assess potential interference from emulsifiers, additional tests included emulsifiers commonly used in intravenous NEs, such as egg yolk phospholipids, soybean phospholipids, Kolliphor HS15, Kolliphor ELP, Tween 20, Tween 80, and sodium deoxycholate were applied [17]. Literature data suggest that emulsifiers may disturb the chromatographic analysis [36,37]. Hence, we decided to verify whether the HPLC method would be useful for analyzing emulsions with different emulsifier compositions. High concentrations of emulsifiers were analyzed, revealing that Tween 80 disrupted the baseline between 5 and 6 min, creating a diffuse, low-intensity peak that interfered with the CBD peak (t_R_ = 5.26 min) (Figure 3G,H). Similarly, Kolliphor ELP affected the baseline but did so beyond the CBD retention time. Thus, the method is unsuitable for matrices containing Tween 80 or, cautiously, Kolliphor ELP unless these emulsifiers’ influence on the assay results is proven negligible. For the remaining emulsifiers tested, no interference was observed.

##### Calibration Curves

The relationship between peak area (P_i_) and CBD concentration was analyzed using ordinary least squares (OLS) regression (Figure S2). With a correlation coefficient of r = 0.9998, the method demonstrated linearity from ~2 to ~80 μg/mL (*n* = 18). The regression analysis showed no statistically significant intercept. Thus, the relationship P_i_ = f(c) was expressed as y = (13.65 ± 0.13)x. The limits of detection (LOD) and quantification (LOQ) calculated by ordinary least squares methods (OLS) were 0.56 μg/mL and 1.70 μg/mL, respectively.

##### Accuracy and Precision

The method’s precision and accuracy were validated through intra- and inter-day assessments for CBD in CBD-enriched hemp oils and NEs at various concentrations (Table 3). For hemp oils (with and without α-tocopherol), CBD concentrations of ~5 μg/mL, ~20 μg/mL, and ~45 μg/mL (*n* = 6) were tested, corresponding to ~0.24%, ~1.0%, and ~2.24% CBD in oils. For NEs, CBD concentrations of ~2.5 μg/mL, ~10 μg/mL, and ~22 μg/mL (*n* = 6) correspond to ~0.025%, ~0.100%, and ~0.225% CBD in NE. In all cases, percentage relative error (RE%) results for intra- and inter-day accuracy were less than 2.3% (acceptance criteria ≤ 5%), and values of relative standard deviation in percent (RSD%) for precision below 1.8% (acceptance criteria ≤ 3%). The values of the obtained parameters are at a satisfactory level, which confirms the accuracy and precision of the method.

##### Linearity

The suitability of the developed method for determining CBD in oils and NEs was further confirmed by plotting the CBD peak area and declared CBD content in hemp oils and NEs, and by a graph of the determined CBD content as a function of the declared content.

Regression analysis of CBD peak area from the declared API (active pharmaceutical ingredient) content was described by the equations as follows:

- CBD-enriched oil without α-tocopherol: y = (13.64 ± 0.73)x; r = 0.9987,
- CBD-enriched oil with α-tocopherol: y = (13.95 ± 0.51)x; r = 0.9994,
- NEs: y = (13.63 ± 0.10)x; r = 0.9999.

Therefore, the values of a (slope) for relationships between the determined CBD content as a function of the declared content of c_det_ = *a* × c_dec._ are: 1.022 ± 0.024 (CBD-enriched oil), 1.003 ± 0.016 (CBD-enriched oil containing α-tocopherol), and 1.007 ± 0.008 (NEs). They belong to the range of 0.998–1.046, 0.987–1.019, and 0.999–1.015, respectively, which includes the value of 1, which indicates an accuracy corresponding to satisfactory recovery.

The results confirm the developed method’s suitability, accuracy, and precision for CBD analysis across diverse formulations.

## 3. Materials and Methods

### 3.1. Materials

Cannabidiol was purchased from Medcolcanna Organics Inc. (Calgary, AB, Canada), refined hemp oil from Molpharma (Ustroń, Poland), Glycerol from PPH Micropharm (Zabierzów, Poland), SLS, and α-Tocopherol from Sigma-Aldrich (St. Louis, MO, USA). The soybean phospholipids (Lipoid P 75) and the egg yolk phospholipids (Lipoid E 80) were very kindly gifted by Lipoid GmbH (Ludwigshafen, Germany). Kolliphor HS15 and ELP; Tween 20, and 80; sodium deoxycholate were purchased from Sigma-Aldrich (St. Louis, MO, USA). Water for injection was purchased from B. Braun Melsungen AG (Melsungen, Germany). All organic solvents used in the studies were of analytical or high-performance liquid chromatographic grade and were purchased from Avantor Performance Materials Poland (Gliwice, Poland).

### 3.2. CBD-Enriched Hemp Oil Preparation

The CBD-enriched hemp oil was prepared in three concentrations (0.5%, 1%, and 1.5%), and each concentration was prepared with and without adding α-tocopherol. For this purpose, the weighted amount of refined hemp oil was added to the appropriate amount of CBD and/or α-tocopherol. The concentration of α-tocopherol in CBD-enriched hemp oils was 1% (50 mg α-tocopherol/5 g oil). All samples were stirred on a magnetic stirrer at 500 rpm for 30 min, protected from light, at room temperature to ensure complete dissolution.

### 3.3. Equipment

The chromatographic analysis was performed on an Agilent 1220 Infinity LC System (Agilent Technologies, Bolinem, Germany) equipped with a dual-channel gradient pump with degasser, a vial autosampler, a multicolumn thermostat, and a diode array detector (DAD). The final chromatographic conditions were:

- stationary phase: LiChrosorb RP18 column, 125 × 4 mm, 5 μm (Merck, Darmstadt, Germany),
- mobile phase: 10% acetonitrile (A)–acetonitrile (B), gradient elution (0–8 min 56% B → 80% B; 8–9 min 80% B → 56% B; 9–12 min 56% B), mobile phase flow rate: 1.5 mL/min
- autosampler temperature: 15 °C,
- column temperature: 40 °C,
- injection volume: 10 μL,
- analytical wavelength: 230 nm.

Photodegradation was carried out in a photostability chamber—the Atlas Suntest CPS+ (Atlas, Mount Prospect, IL, USA), which was equipped with a temperature control system (25–100 °C), 1500 W air-cooled xenon lamps, and direct setting and control of irradiance in the wavelength range 300–800 nm (filter Solar ID65). The photodegradation was performed at 25 ± 2 °C.

An accelerated stability test at elevated temperature (60 ± 2 °C) and relative air humidity (75 ± 5% RH) was performed in a Binder KBF 240 climatic chamber (Binder, Germany). The working range of the climatic chamber is from 0 °C to 70 °C, and the relative humidity ranges from 10% to 80%.

The pH of the tested samples or buffers was measured in triplicate with a Mettler-Toledo AG SevenCompact^TM^ pH/ion S220 pH-meter (Schwerzenbach, Switzerland) at room temperature.

The values of MDD, PDI, ZP, and peak sizes I and II of prepared NEs were determined using Zetasizer Nano ZS (Malvern Instruments, Malvern, UK).

### 3.4. CBD-Enriched Oils Stability Study

CBD-enriched oils with or without α-tocopherol were placed in 20 mL sealed vials for stability studies. The studies were performed in four different conditions: at room temperature (22 ± 2 °C) with and without light access, at refrigerator conditions (the temperature of 4 ± 2 °C), and in an aging chamber at 60 ± 2 °C and 75 ± 5% RH (without light access). The samples were taken at specified time intervals. Then, the weighted samples of CBD-enriched oils (about 20 mg) were mixed with 1.0 mL of DCM, diluted to 10.0 mL with methanol, and injected into the HPLC column. 

### 3.5. Development of CBD-Enriched Intravenous Nanoemulsions

The CBD-enriched hemp oils were used to develop intravenous NEs. Briefly, the CBD-enriched oils containing 1% of α-tocopherol were homogenized with the aqueous phase consisting of glycerol, soybean phospholipids, and water for injection using a two-step homogenization method involving mechanical homogenization followed by high-pressure homogenization. The composition of the CBD-enriched intravenous NEs is presented in Table 4.

The aqueous phase was prepared by dissolving phospholipids and glycerol in water for injection using a heating magnetic stirrer at 500 rpm, 70 °C for 30 min. The oil phases consisting of CBD, α-tocopherol, and hemp oil were mixed using a magnetic stirrer at 500 rpm, protected from light, for 20 min at room temperature and further for 10 min at 50 °C. The oil phase was added dropwise into the aqueous phase during the mechanical homogenization. This process was performed using homogenizer Unidrive X 1000 D (Ingenieurburo Cat, M. Zipperer GmbH, Ballrechten-Dottingen, Germany) set to 10,000 rpm for 30 min. The resulting course emulsions were then homogenized using a high-pressure homogenizer under 1000 bar in 10 cycles (GEA PandaPLUS 2000, GEA Niro Soavi, Parma, Italy) to obtain the final NEs. In the last step, the developed NEs were adjusted to a pH of 8.0 using 0.1 M sodium hydroxide and filtered using a 0.2 µm cellulose filter to obtain a sterile formulation. Developed NEs were stored for 21 days at 4 ± 2 °C.

### 3.6. Characterization of CBD-Enriched Intravenous Nanoemulsions

The MDD, PDI, peak size I and II, and ZP of developed NEs were determined using Zetasizer Nano ZS (Malvern Instruments, UK). Briefly, 100 µL of NEs were diluted in 9.9 mL of water for injection, transferred to a polycarbonate cuvette, and placed in a detention cell. To assess the significance of changes in MDD after filtration and after 3 weeks of storage, the ANOVA test was performed with a significance level of *p* < 0.05. Samples after sterilization filtration were compared to those just after preparation, and the samples after 3 weeks of storage were compared with those after filtration sterilization. Moreover, the content of CBD was assessed in oils used for the NEs preparation, in NEs after preparation, after filtration sterilization, and after 3 weeks of storage in a refrigerator.

### 3.7. HPLC-DAD Method Development

#### 3.7.1. HPLC Method Development and Optimization

Several isocratic and gradient conditions were investigated to develop and optimize an analytical HPLC method for quantitatively determining CBD and its separation from degradation products, impurities, and excipients.

The samples of CBD and CBD-enriched hemp oils were subjected to degradation under various environmental conditions. CBD was exposed to temperature (room temperature or 37 ± 2 °C), pH, and light. CBD was subjected to photodegradation in ethanol and thermal degradation in in a mixture of 0.8% ethanol and aqueous solutions with 1% SLS (in 0.1 M NaOH, HCl, phosphate buffer pH 6.8 and 7.4 at 37 ± 2 °C). Approximately 2.5 mg of CBD was accurately weighed and dissolved in 25.0 mL of 96% ethanol (degradation in ethanol). Approximately 2.5 mg or 5 mg of CBD was accurately weighed and dissolved in 0.2 mL of 96% ethanol and then adjusted to 25.0 mL with an appropriate solution (1% SLS; 1% SLS in 0.1 mol/L NaOH or 0.1 mol/L HCl; 1% SLS in phosphate buffer). If the study was conducted at an elevated temperature (37 ± 2 °C), the added medium was preheated to the required temperature. The samples were mixed, and 1.0 mL was withdrawn (sample at t = 0 min). The remaining test solution was placed in an incubator, Suntest aging chamber, or left at room temperature (22 ± 2 °C). At specified intervals, 1.0 mL of the test solution was withdrawn, neutralized, and cooled in a mixture of ice and water (if necessary), and injected into the column.

In turn, hemp oil and CBD-enriched hemp oils with or without α-tocopherol were placed in 20 mL sealed vials and stored in an aging chamber at 60 ± 2 °C and 75 ± 5% RH (without light access). The samples were prepared by mixing 20 mg of degraded CBD oil with 1.0 mL of DCM and diluted to 10.0 mL with methanol. The prepared samples were then injected into the column.

After establishing appropriate chromatographic separation conditions, a robustness test was carried out, and the stability of the alcoholic CBD solution (standard solution) was checked. Robustness was evaluated by changing the following operational parameters of the developed chromatographic method: column and autosampler temperature, flow rate, injection volume, and wavelength. Three ethanolic test solutions (~30 μg of CBD/mL of ethanol) prepared from a model mixture (~50% CBD in the model mixture) were analyzed. The concertation of CBD in the model mixtures was calculated using the standard sample (~60 μg of CBD/mL of alcohol). The stability of the analyte (~40–60 μg of CBD/mL of alcohol) stored in a refrigerator (4 ± 2 °C, 5 days), at an autosampler temperature (15 °C, 24 h) and room temperature (22 ± 2 °C, 24 h), without access to light, was proved.

#### 3.7.2. HPLC Method Validation

The chromatographic conditions considered optimal during the method development were subjected to the validation process, including parameters such as selectivity, linearity, LOD and LOQ, intra- and inter-day precision, accuracy, and system suitability. The selectivity of the HPLC method was evaluated by ensuring the complete separation of CBD from its degradation products, oil peaks, and solvents/ingredients.

To achieve this, CBD underwent photodegradation in ethanol and thermal degradation in ethanolic and aqueous solutions; CBD-enriched oils were exposed to various factors, including elevated temperature (at 60 ± 2 °C and 75 ± 5% RH, 4 weeks, in closed vials, without light) and light (daylight, 8 weeks; room temperature); the tested NEs were exposed to elevated temperature (40 ± 2 °C for 10 days, 80 ± 2 °C for 24 h and 134 ± 2 °C for 12 min). Additionally, samples of CBD (4 μg of CBD/mL) with various emulsifiers were checked. The samples were prepared by mixing a methanolic solution of CBD with methanolic solutions of emulsifiers, DCM, and methanol with a final concentration of 4 μg of CBD/mL and 0.05% of emulsifiers. Samples prepared during the selection of chromatographic conditions were used to test selectivity.

##### System Suitability Test

The system suitability was assessed based on the injection of a standard solution six times (CBD concentration c = ~55 μg/mL) and determining the number of theoretical plates (N), the symmetry factor, as well as the repeatability of the peak position (t_R_) and peak area.

##### Calibration Curves

Calibration curves were established by preparing samples from ~2 to ~80 μg/mL (*n* = 6). During the validation procedure, stock solutions (~1 mg CBD/mL methanol) were utilized, which were subsequently diluted in methanol to achieve concentrations within the calibration ranges. Three separate series of calibration standards were prepared. The linear regression parameters of the plots P = f(c), where P—peak area, c—CBD concentration [μg/mL], were calculated using OLS. Moreover, limits of detection LOD = 3.3 *S_b_*/*a* and limits of quantitation LOQ = 10 *S_b_*/*a* were determined based on the parameters of OLS (*S_b_*—the standard deviation of intercept, *a*—the slope of calibration curve.

##### Linearity

To determine the linearity of the method, CBD-enriched oils with and without α-tocopherol were prepared at the following CBD concentration levels: approximately 0.24%, 0.5%, 0.8%, 1%, 1.2%, 1.5%, and 2.24%. These samples were prepared by weighing about 6 mg, 12.5 mg, 20 mg, 25 mg, 30 mg, 37.5 mg, and 56 mg of CBD, adding 2.5 g of hemp oil and for CBD-enriched oils containing α-tocopherol (25 mg of α-tocopherol per 2.5 g of hemp oil). All samples were stirred on a magnetic stirrer to ensure complete dissolution at room temperature.

Analogously, an emulsion with a CBD concentration of 0.225% and a blank emulsion were prepared to assess the linearity of the CBD determination in the emulsions. Then, a series of emulsions with concentrations of ~2.5 μg/mL–~22 μg/mL were prepared by diluting it with blank emulsion.

Subsequently, approximately ~15–20 mg of the appropriate CBD-enriched oil or ~90–100 mg of NEs were mixed with 1.0 mL of DCM and diluted to 10.0 mL with methanol. Three samples were analyzed for each concentration. The samples of the analyzed compounds were protected from light. Then, CBD peak area versus theoretical API content and the determined CBD content as a function of its declared content plots were created, and statistical parameters were calculated for both dependencies above.

##### Precision and Accuracy

The CBD oils samples for precision and accuracy intra- and inter-day were prepared according to the procedure described for linearity at three concentrations: ~5 μg/mL, ~20 μg/mL, and ~45 μg/mL, and for NEs also at three concentrations: ~2.5 μg/mL, ~10 μg/mL, and ~22 μg/mL (*n* = 6).

The results of precision were expressed as RSD% and calculated from six samples for each tested concentration:

(1) $$RSD\%=\frac{SD}{X_{av}}\times 100\%$$

where SD—standard deviation, X_av_— average value.

Accuracy (*n* = 6) was calculated according to the following formula:

(2) $$Accuracy=\frac{{|c}_{t}-c_{d}|}{c_{t}}\times 100\%$$

where *c_d_*—is determined from the ordinary linear equation; *c_t_*—is the theoretical concentration. Different analysts performed the intra- and inter-day precision and accuracy in a time interval.

## 4. Conclusions

In this study, we developed a novel intravenous NEs based on CBD-enriched hemp oil. Lipid emulsions hold significant potential in clinical practice, not only as components of parenteral nutrition but also as delivery systems for poorly soluble drugs administered intravenously, orally, and intranasally [38,39,40,41]. The formulated NEs show great promise for further development and comprehensive in vitro and in vivo investigations. Additionally, the developed and validated HPLC-DAD method allows for determining the CBD content in the presence of degradation products across various matrices, including alcoholic solutions, water, and oil phases. This method was successfully applied to assess the stability of CBD-enriched hemp oils, which were subsequently utilized in preliminary studies to develop intravenous NEs. As a part of the NE characterization process, we evaluated the effects of homogenization and sterilization on CBD content. Furthermore, our findings highlighted that certain emulsifiers used in NE formulations may interfere with CBD analysis, underscoring the importance of careful method selection depending on the excipient presented in the formulation. We believe that the proposed method could be useful as a reliable standard quality control procedure for commercial drug testing laboratories, pharmaceutical industries, and research laboratories. This standardized approach would ensure the quality assessment of CBD oils, and CBD-containing NEs.

## Figures and Tables

**Table 1:** The results of stability tests of CBD-enriched hemp oils in various conditions.

Storage Conditions	Time	CBD Oils			CBD Oils with α-Tocopherol		
		0.5%	1.0%	1.5%	0.5%	1.0%	1.5%
		Mean CBD content ± SD [%], *n* = 3					
Room temperature with light	0	100 ± 0.7	100 ± 0.6	100 ± 0.6	100 ± 1.2	100 ± 1.0	100 ± 0.5
	2 weeks	96.4 ± 0.6	97.0 ± 0.4	95.2 ± 0.5	94.1 ± 1.7	96.8 ± 1.3	96.6 ± 1.1
	8 weeks	85.8 ± 1.7	89.9 ± 1.2	94.4 ± 0.4	83.6 ± 1.6	89.0 ± 1.2	92.1 ± 1.1
Room temperature, no light	0	100 ± 0.7	100 ± 0.6	100 ± 0.6	100 ± 1.2	100 ± 1.0	100 ± 0.5
	2 weeks	98.4 ± 0.9	96.2 ± 0.8	95.3 ± 0.2	96.4 ± 0.4	97.0 ± 1.1	96.8 ± 0.4
	8 weeks	94.4 ± 1.1	91.0 ± 0.2	95.4 ± 0.2	89.6 ± 1.0	95.1 ± 1.0	94.5 ± 1.4
Refrigerator	0	100 ± 0.7	100 ± 0.6	100 ± 0.6	100 ± 1.2	100 ± 1.0	100 ± 0.5
	2 weeks	98.9 ± 0.7	99.4 ± 0.6	98.2 ± 0.7	95.8 ± 2.9	98.5 ± 0.9	97.8 ± 0.8
	8 weeks	95.2 ± 1.4	96.4 ± 0.4	96.4 ± 0.3	95.1 ± 0.2	95.9 ± 1.7	97.2 ± 0.5
60 ± 2 °C, 75 ± 5% RH closed vial	0	100 ± 0.6	100 ± 0.9	100 ± 0.3	100 ± 1.0	100 ± 3.5	100 ± 1.4
	2 weeks	52.4 ± 1.2	42.4 ± 1.0	55.7 ± 0.5	75.1 ± 7.2	70.2 ± 2.4	74.5 ± 0.6
	4 weeks	20.9 ± 0.8	15.9 ± 0.1	26.4 ± 0.3	40.3 ± 2.6	34.2 ± 0.6	33.6 ± 0.7
Thermal sterilization	before	100 ± 0.6	100 ± 0.9	100 ± 0.3	100 ± 1.0	100 ± 3.5	100 ± 1.4
	after	89.5 ± 0.8	90.6 ± 1.0	90.9 ± 0.4	96.1 ± 3.0	96.0 ± 0.5	96.7 ± 0.2

SD–standard deviation.

**Table 2:** The characteristics of prepared nanoemulsions.

Sample	MDD [nm]	PDI	Peak Size I [nm]	Peak Size II [nm]	ZP [mV]	CBD Content [%]
CBD-enriched oils with α-tocopherol used for NEs preparation						
Oil 1	n/a	n/a	n/a	n/a	n/a	100.9 ± 0.8 *
Oil 2	n/a	n/a	n/a	n/a	n/a	100.1 ± 1.3 *
Oil 3	n/a	n/a	n/a	n/a	n/a	100.4 ± 1.7 *
CBD-loaded nanoemulsions just after preparation						
NE 1	193.2 ± 1.5	0.13 ± 0.00	226.5 ± 1.5	−	−51.2 ± 0.7	101.0 ± 0.7 *
NE 2	189.0 ± 1.4	0.12 ± 0.01	215.3 ± 1.8	−	−25.9 ± 0.2	99.7 ± 0.3 *
NE 3	183.4 ± 1.6	0.14 ± 0.02	210.9 ± 2.7	−	−27.8 ± 0.4	98.5 ± 0.7 *
CBD-loaded nanoemulsions after filter sterilization						
NE 1	191.2 ± 1.7 ^a^	0.10 ± 0.01	215.7 ± 5.0	−	−41.6 ± 0.4	98.0 ± 0.4 **
NE 2	186.3 ± 1.1 ^a^	0.14 ± 0.01	212.7 ± 1.3	−	−28.7 ± 0.2	98.0 ± 4.5 **
NE 3	181.0 ± 2.0 ^a^	0.12 ± 0.02	204.5 ± 4.5	−	−28.4 ± 0.2	98.0 ± 0.3 **
CBD-loaded nanoemulsions after 3 weeks of storage at 4 ± 2 °C						
NE 1	193.4 ± 1.4 ^a^	0.13 ± 0.01	221.8 ± 2.8	−	−42.3 ± 0.5	97.8 ± 0.8 ***
NE 2	181.7 ± 1.7 ^b^	0.11 ± 0.01	207.9 ± 1.8	−	−23.7 ± 0.5	100.4 ± 1.8 ***
NE 3	178.8 ± 0.5 ^a^	0.13 ± 0	200.9 ± 0.6	−	−27.7 ± 0.4	100.5 ± 0.5 ***

^a^—statistically insignificant changes, ^b^—statistically significant change *p* < 0.05, *—CBD content is expressed as a percentage of theoretical content in the formulation, **—CBD content is expressed as a percentage of determined content after preparation or *** after filtration sterilization, n/a—not applicable, MDD—mean droplet diameter, PDI—polydispersity index, ZP—zeta potential. Three measurements were performed for each sample.

**Table 3:** The precision and accuracy of the method of determining cannabidiol (CBD) in CBD-enriched oil (CBD oil), CBD-enriched oil containing α-tocopherol (CBD+E oil), and NEs.

	Precision/Recovery			
	Intra-Day			Inter-Day
	Series I *n* = 6		Series II *n* = 6	Series I-II *n* = 12
	Concentration [μg mL^−1^]	RSD [%] RE [%]	RSD [%] RE [%]	RSD [%] RE [%]
CBD oil	~5	0.93 0.28	1.05 0.41	0.95 0.34
	~20	1.57 −0.61	1.75 −0.74	1.60 −0.89
	~45	0.79 2.29	0.84 −0.02	1.45 1.13
CBD+E oil	~5	0.61 0.18	1.38 −0.20	1.04 −0.17
	~20	0.68 −1.08	0.63 −0.58	0.68 −0.89
	~45	1.42 −0.29	0.62 −0.94	1.09 −0.62
NEs	~2.5	0.55 −1.50	1.11 −0.70	0.93 −1.10
	~10	0.74 −1.06	0.73 0.37	1.02 −0.34
	~22	0.59 0.70	1.06 −0.04	0.90 0.33

**Table 4:** Composition of CBD-loaded intravenous nanoemulsions.

Ingredients	NE1	NE2	NE3
	Content [g]		
Oil phase			
CBD	0.05	0.10	0.15
α-Tocopherol	0.10	0.10	0.10
Hemp oil (refined)	10.0	10.0	10.0
Aqueous phase			
Glycerol	2.5	2.5	2.5
Soybean phospholipids	1.2	1.2	1.2
Water for injection	86.15	86.10	86.05

## Data Availability

Data are contained within the article and Supplementary Materials, and a more detailed dataset is available from the authors on request.

## Supplementary Materials

The following supplementary materials can be downloaded at: https://www.mdpi.com/article/10.3390/molecules30020278/s1, Figure S1. Chromatogram of NE based on hemp oil not enriched in CBD (placebo) (A); chromatogram of NE based on CBD-enriched hemp oil (B). All injected solutions were prepared based on methanol and dichloromethane. CBD—cannabidiol; Figure S2. Plots of CBD peak area vs. CBD concentration; Table S1. Results of the robustness test; Table S2. Results of the suitability test.
