# Supplementary material for: Novel Intravenous Nanoemulsions Based on Cannabidiol-Enriched Hemp Oil—Development and Validation of an HPLC-DAD Method for Cannabidiol Determination
Source: Molecules. 2025 Jan 12;30(2):278. doi: 10.3390/molecules30020278 (PMC11767503; doi:10.3390/molecules30020278)
Supplement: Supplementary file 1 [file molecules-30-00278-s001.zip › molecules-3363134-supplementary.pdf]

Supplementary Materials for Article

Novel Intravenous Nanoemulsions Based on Cannabidiol-Enriched Hemp Oil—Development and Validation of an HPLC-DAD Method for Cannabidiol Determination

Agnieszka Sobczak *, Piotr Zieliński, Anna Jelińska and Aleksandra Gostyńska-Stawna

Chair and Department of Pharmaceutical Chemistry, Poznan University of Medical Sciences, Rokietnicka 3, 60-806 Poznan, Poland; ajelinsk@ump.edu.pl (A.J.); agostynska@ump.edu.pl (A.G.-S.)
* Correspondence: asobczak@ump.edu.pl

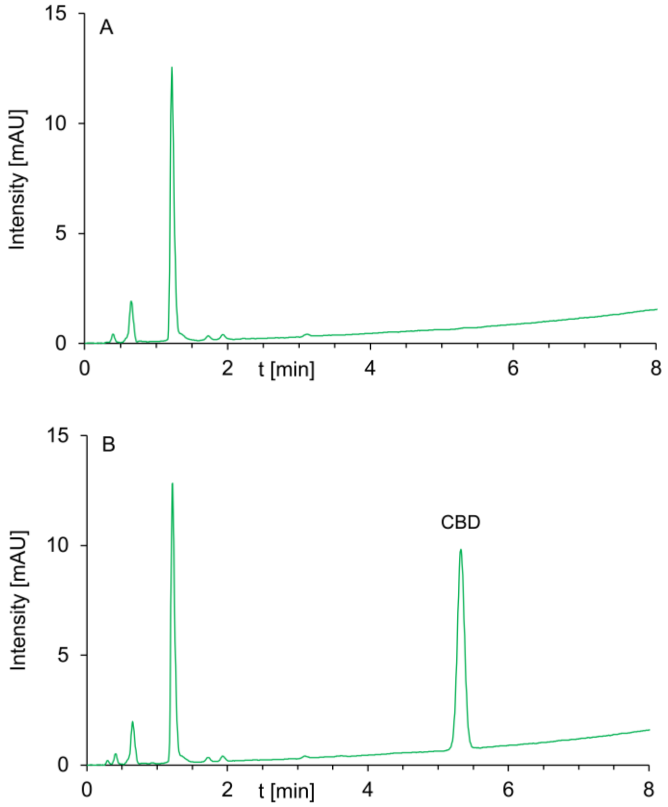

Figure S1. Chromatogram of NE based on hemp oil not enriched in CBD (placebo) (A); chromatogram of NE based on CBD-enriched hemp oil (B). All injected solutions were prepared based on methanol and dichloromethane. CBD – cannabidiol.

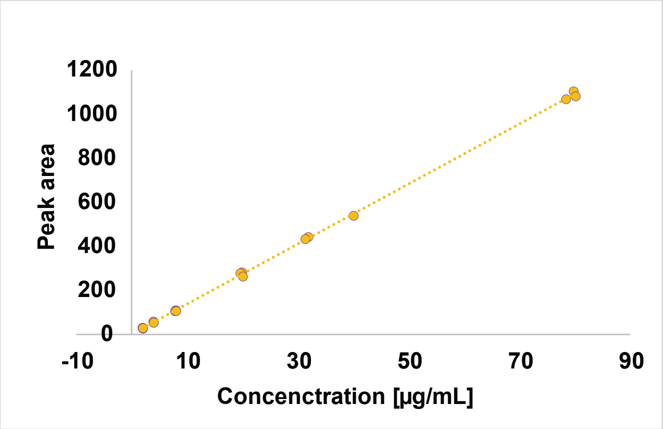

Figure S2. Plots of CBD peak area vs. CBD concentration.

Table S1. Results of the robustness test

Parameter changed intentionally		CBD concentration [%]	X [%]	Peak symmetry
Column temperature	45°C	50.56	0.06	0.944
	40°C	50.59	–	0.929
	35°C	50.66	0.14	0.939
Autosampler temperature	20°C	50.56	0.14	0.945
	15°C	50.59	–	0.929
	10°C	50.52	0.14	0.953
Flow rate	1.65 mL/min	51.01	0.83	0.914
	1.50 mL/min	50.59	–	0.929
	1.35 mL/min	50.57	0.04	0.964
Injection volume	12 µL	50.67	0.04	0.959
	10 µL	50.65	–	0.929
	8 µL	50.70	0.10	0.958
Analytical wavelength	232 nm	50.65	0.00	0.961
	230 nm	50.65	–	0.929
	228 nm	50.68	0.06	0.961

X [%] – change in [%] cannabidiol (CBD) content in the model mixture determined using HPLC conditions with one parameter changed in relation to the result obtained under standard conditions (absolute value).

Table S2. Results of the suitability test

Parameters	Determined parameters	Critical parameters
Retention time t_R [min]	5.26	3%
RSD [%]	0.07	
Peak area	631.7	3%
RSD [%]	0.6	
Number of theoretical plates	11113	N > 8000
Peak symmetry	0.987	0.8-1.5

RSD [%] – relative standard deviation in percent
